# E-Selectin Mediated Adhesion and Migration of Endothelial Colony Forming Cells Is Enhanced by SDF-1α/CXCR4

**DOI:** 10.1371/journal.pone.0060890

**Published:** 2013-04-02

**Authors:** Jie Sun, Yuhua Li, Gina M. Graziani, Lionel Filion, David S. Allan

**Affiliations:** 1 Bone Marrow Transplantation Centre, Medical College of Zhejiang University, Hangzhou, Zhujiang Province, China; 2 Sprott Centre for Stem Cell Research, Regenerative Medicine Program, Ottawa Hospital Research Institute, Ottawa, Ontario, Canada; 3 Departments of Biochemistry, Microbiology and Immunology, University of Ottawa, Ottawa, Ontario, Canada; 4 Department of Medicine, University of Ottawa, Ottawa, Ontario, Canada; University of Padua, Italy

## Abstract

**Objective:**

Endothelial-colony forming cells (ECFCs) can be readily expanded from human umbilical cord blood and can facilitate repair of endothelial injury. E-selectin and SDF-1α are produced following endothelial injury and can regulate endothelial progenitor homing. Mechanisms of vascular repair specific to the mode of injury have not been well described in homogenous cell populations such as ECFCs and are needed for development of more effective vascular repair strategies.

**Methods and Results:**

Lipopolysaccharide (LPS)-induced endotoxic injury to mature human umbilical vein endothelial cells (HUVEC) was compared with hypoxic and radiation injury. E-selectin expression in HUVEC cells is markedly increased (208-fold) following LPS-induced injury and facilitates increased ECFC adhesion and migration function in vitro. SDF-1α expression remains unchanged in LPS-treated HUVEC cells but increases more than 2 fold in fibroblasts undergoing similar endotoxic injury. SDF-1α induces expression of E-selectin ligands on ECFCs and facilitates greater E-selectin-mediated adhesion and migration of ECFCs in a CXCR4-dependent manner. Induction of E-selectin expression in HUVECs following hypoxic or radiation injury is negligible, however, while SDF-1α is increased markedly following hypoxia, highlighting injury-specific synergism between mediators of vascular repair.

**Conclusion:**

E-selectin mediates adhesion and migration of ECFCs following endotoxic endothelial injury. SDF-1α augments E-selectin mediated ECFC adhesion and migration in a CXCR4-dependent manner.

## Introduction

Vascular endothelial injury underlies many medical conditions including sepsis, occlusive vascular disease affecting the renal, cardiovascular and cerebrovascular systems, microangiopathies such as thrombotic thrombocytopenic purpura, vasculitic disorders including autoimmune conditions, and graft versus host disease that can complicate blood stem cell transplantation. Vascular injury can be systemic or isolated to a single organ and may be caused by various insults including ischemia, endotoxic damage related to infection, immune-mediated or following treatments such as chemotherapy and radiation. Serious organ dysfunction can result which is often irreversible. New treatments are needed to limit vascular damage and facilitate timely and complete repair to reduce the morbidity and mortality associated with vascular injury and to lessen the burden on health care resources.

Since Asahara *et al* [1] first described endothelial progenitor cells (EPCs) as a subset of CD34+ cells isolated from bone marrow, endothelial progenitors and related cell types have been characterized from bone marrow, peripheral blood, or other tissues and contribute to endothelial repair and neovascularization in several models of endothelial damage [2]. Circulating endothelial progenitors represent a heterogeneous population of myeloid and monocytic cells derived from bone marrow and may include rare cells with specific endothelial precursor function.[3], [4] Early outgrowth EPCs are a heterogenous population of adherent mononuclear cells from peripheral blood or umbilical cord blood that emerges *in vitro* under angiogenic culture conditions after approximately 5 – 7 days.[5] The precise identity of various endothelial progenitor cell populations remains under active study. In contrast, *ex vivo* expansion of endothelial colony forming cells (ECFCs) from peripheral blood or umbilical cord blood provides a homogenous population of endothelial-like cells with a high proliferative capacity, blood-forming function and therapeutic potential in several models of vascular injury. [5]–[7] Moreover, cells that contribute to vascular repair can be differentiated from CD34+ haematopoietic stem cells and can be mobilized into peripheral blood following vascular injury [8]–[13], or following administration of angiogenic cytokines including VEGFA [14], G-CSF [15], GM-CSF [16], EPO [17], [18] and plerixafor, a CXCR4 antagonist. [19]

Vascular repair involves the mobilization and homing of appropriate cell types from their steady state niches to zones of vascular injury. Homing is a multi-step process that involves migration and adhesion of cells to denuded extracellular matrix (ECM) under the regulation of chemokines and their receptors to facilitate differentiation into mature endothelial cells and to form new microvessels.[20] Several cell types appear to be involved in this repair process and recruitment and adhesion of cells to the area of injury likely occurs in a coordinated step-wise manner through the action of numerous chemokines and receptors. [21]–[29] Homing is considered an essential step for neovascularization in postnatal life.

SDF-1α has been widely studied as a central chemokine involved in vascular repair and is widely expressed by numerous tissues. Its secretion increases from damaged tissues under different kinds of vascular endothelial injuries including acute ischemic kidney injury [30]; limb ischemia [7]; toxic liver damage [31] and total body irradiation [32]. SDF-1α/CXCR4 signaling is considered to play a central role in mobilizing endothelial progenitors from bone marrow [33], [34]. Recently SDF-1α was also shown to participate in homing of endothelial progenitors by up-regulating their adhesion and migration. [35] SDF-1α was shown to increase migration of endothelial progenitors to injured tissues through up regulation of β_2_ integrins on their cell surface. [23] In addition, E-selectin is an adhesion molecule which was recently found to regulate endothelial progenitor homing [36] and appears to work together with SDF-1α [37]. However, the precise mechanisms by which SDF-1α and E-selectin exert their effects on homing of endothelial progenitors have not been fully elucidated. Furthermore, the effect of E-selectin and the role of SDF-1α have not been addressed in homogenous cell populations such as ECFCs. In this report, we describe *in vitr*o studies of endotoxic injury to endothelial cells and the role of E-selectin in the migration and adhesion of ECFC cells. We also describe how SDF-1α can augment cell surface E-selectin ligands on ECFCs which may underscore mechanisms to enhance vascular repair function. In addition, we provide insight on specific chemokine responses following different types of vascular injury and how E-selectin mediated vascular repair may be injury-specific.

## Methods

### Ethics Statement

Human umbilical cord blood samples were obtained from healthy donors at The Ottawa Hospital and Queensway Carleton Hospital following written informed consent in accordance with protocols approved by the Ottawa Hospital Research Ethics Board.

### Isolation and identification of endothelial colony forming cells (ECFCs)

Mononuclear cells (MNCs) from human umbilical cord blood were isolated by Ficoll density gradient centriguation as previously described [38]. To expand ECFCs, 1.0 × 10^7^ MNCs were plated on Costar CellBIND Surface 6-well plates (Corning) with EGM2 growth media (Lonza) supplemented with 10% fetal bovine serum (FBS) (GIBCO) and Singlequot growth factors (Clonetics). Serum-supplemented media was changed every day during the first week and then every other day. Non-adherent cells were discarded with each change of media. Colonies with cobblestone morphology appearing between days 9 to 16 were isolated and re-plated in EGM2 media with 10% FBS and Singlequot growth factors. Only ECFCs from passage 2 to 4 were used for subsequent experiments.

ECFCs were characterized by resuspending 1 × 10^6^ cells in 2% FBS and performing flow cytomrtry using the antibodies with the following specificity: anti-CD133-PE (MACS), anti-CD34-FITC (MACS), anti-CD45-APC or ECD (MACS), anti-CD31-FITC (MACS) and anti-VEGFR2-APC (R&D Systems) antibodies. Flow cytometric analysis was performed using the CyAn ADP9 analyser (Beckman Coulter) and interpreted using Kaluza software (Beckman Coulter).

For the detection of acetylated-LDL (Ac-LDL) uptake, cells were incubated with EGM2 media containing 10 µg/mL Ac-LDL for 4 hours at 37 °C, then fixed with 4% paraformaldehyde (Biomedical Technologies Inc) for 10 minutes and stained with 4′,6-diamidino-2-phenylindole (DAPI) (Pierce). To detect von Willebrand Factor (vWF), cultured ECFCs were first permeabilized by incubating with 0.1% Triton X-100 for 10 minutes, fixed and then blocked with phosphate-buffered saline (PBS) supplemented with 10% FBS for 30 minutes. Cells were incubated with rabbit anti-vWF antibody (Santa Cruz) for 2 hours, and then were incubated with Alexa Fluor 488 goat anti-rabbit antibody (Invitrogen) for 1 hour and stained with DAPI. To detect CD14 and CD133 surface markers, anti-CD14-FITC (BD Pharmingen) and anti-CD133-PE antibodies were used. Flouresence microscopy was performed using the Zeiss Observer Z1 microscope (Carl Zeiss Ltd).

### Cell lines

Human Umbilical Vein Endothelial Cells (HUVEC) were obtained from the supplier (Lonza) and cultured according to instructions. Briefly, HUVECs were cultured in EGM2 media (Lonza) supplemented with 10% FBS (GIBCO). Cells were not utilized for any experiments beyond the third passage. MRC-5 cells (human fetal lung fibroblasts, American Type Culture Collection) were generously provided by Dr. Lisheng Wang (University of Ottawa) and cultured in low glucose Dulbecco's Modified Eagle Media (DMEM, GIBCO) supplemented with 10% FBS.

### Endotoxic, hypoxic and radiation-induced cellular injury

Endotoxic cellular injury was performed as previously described [39], [40]. Briefly, lipopolysaccharide (LPS) (Sigma) 100ng/ml and soluble CD14 (R&D systems) 1.0 µg/ml were added for 2 hours to HUVEC cells grown at 80% confluence. Injury levels were evaluated by measuring lactate dehydrogenase (LDH) activity in accordance with the instructions provided by the manufacturer of the LDH Activity Assay (Biovison). Aliquots (25 µl) of the culture supernatant were used for LDH detection at various time points following the induction of cellular injury. The conditioned media from cells undergoing endotoxic injury was collected and centrifuged at 9300 × g for 10 minutes (Thermo Electron Corporation) and stored at −80^o^C for use in subsequent assays of ECFC function. In some experiments, we investigated the role of depleting soluble E-selectin produced in the conditioned media of injured HUVEC cultures by adding 1 µg/ml anti-CD62E (BD Pharmingen) to injured HUVEC cells for 1 hour and in other experiments, we blocked the E-selectin ligand CD44 on ECFCs with 1 µg/mL rat anti-human CD44 antibody (HCAM-IM7, Santa Cruz) for 1 hour prior to adhesion or migration assays.

Hypoxic injury was induced by incubating HUVEC cells or MRC-5 cells grown to 80% confluence at 37 °C with 5% CO_2_, 94% N_2_, and 1% O_2_ in a multi gas autoflow CO_2_ water-jacketed incubator (NuAire) for 3, 6, and 24 hours, in accordance with previous reports [41], [42]. Levels of cellular injury were assessed using the LDH assay as described above.

Radiation-induced cellular injury was performed as previously described [43], [44]. Briefly, 400 cGy X-ray irradiation was applied by a HF-320 irradiator (Pantak) at a dose rate of 40 cGy/min to cultures of HUVEC or MRC-5 cells grown to 80% confluence. Cellular injury was assessed using the LDH assay as described above.

### Evaluation of ECFC adhesion and migration

ECFC homing function was evaluated using *in vitro* tests of adhesion and migration. To assess adhesion, ECFCs were serum-deprived in EGM2 media overnight and then 5 × 10^4^ cells plated on fibronectin-coated 24-well (2.0 cm^2^) plastic dishes (Fisher Scientific) in duplicate and incubated for 20 minutes at 37 °C in the presence of conditioned media (500 µl) from injured or control HUVEC or MRC-5 cells or adhesion buffer (0.5% BSA in EGM2 media). The wells were then washed three times with 0.5 ml adhesion buffer to remove non-adherent cells. Adherent ECFCs were stained with DAPI as described above and then counted in five random fields per well at 50x magnification. Results are reported as the mean number of attached cells per field ± SEM. In some experiments, ECFCs were plated on HUVEC cells cultured in 24-well plates to 90% confluence and serum-deprived overnight. ECFCs were incubated with 10 µg/mL Ac-LDL for 4 hours prior to plating on HUVEC cells to allow identification of ECFC cells.

In some experiments, ECFC adhesion was assessed by adding 2 – 4 µg/ml recombinant human E-selectin (R&D systems) or 50 – 200 ng/ml human recombinant SDF-1α (Prospec) in adhesion buffer to serum-deprived ECFC cultures for 2 – 12 hours prior to performing the adhesion assay. As a positive control, ECFCs were incubated with 100 ng/ml VEGFA (R&D Systems) prior to performing the adhesion assay. To block the E-selectin ligand CD44, 5 × 10^4^ ECFC cells were incubated with 1:100 (v/v) rat anti-human CD44 antibody (HCAM-IM7, Santa Cruz) for 1 hour. To block CXCR4 on ECFC cells, 5 µg/ml plerixafor (Amgen) was added to ECFC cultures 30 min prior to the adhesion assay.

To assess ECFC migration, 5 × 10^5^ cells were suspended in 200 µl adhesion buffer and plated on the upper part of a Boyden chamber (ThinCerts transwells 8 µM pore, Greiner Bio-One). Cells were incubated at 37^o^C for 4 hours to permit migration through the pores in the membrane toward the lower chamber containing candidate mediators of ECFC migration in 500 µl adhesion buffer. To enumerate ECFC migration, the underside of the membrane was stained with Diff-Quick (BD Pharmingen) and cells were counted in five random high powered fields (hpf) at 100x magnification using Image J software (Image J is publicly available software provided by the US National Institutes of Health).

### Real-time polymerase chain reaction analysis

Total RNA (0.5 µg) isolated from cells with RNeasy Mini Kit (Qiagen) was used for cDNA synthesis by M-MuLV reverse transcriptase (Biolabs). cDNA transcripts were then amplified by real-time polymerase chain reaction (RT-PCR) with specific primers according to the manufacturer's instructions. Briefly, the reactions contained SYBR Green I mix (Invitrogen), 10 pmol of both forward and reverse primers, and cDNA corresponding to 5 ng total RNA. All reactions were performed in duplicate. The reactions were subjected to 45 cycles of PCR amplification (95 °C for 10 seconds and 60 °C for 20 seconds, 72 °C for 34 seconds in Rotor-GeneQ (Qiagen) detection system. All results were normalized to GAPDH mRNA. The primers used are provided in the Table 1.

**Table 1:** PCR primers.

Gene	Primers	Length (bp)
E-selectin	5′primer: CCGTCCGCCAGCCTCAGAAT	214
	3′primer: TAGCCTCGCTCGGGGTTGGAC	
VCAM-1	5′primer CCATTTGACAGGCTGGAGAT	201
	3′primer TACTGTGGGCACAGAATCCA	
ICAM-1	5′primer CAGAGGTTGAACCCCCACAGT	203
	3′primer TCTGAGACCTCTGGCTTCGT	
GAPDH	5′primer: CCACCCAGAAGACTGTGGAT	423
	3′ primer: CCCTGTTGCTGTAGCCAAAT	
CD44	5′ primer: CGCCCAGGGATCCTCCAGCT	106
	3′ primer: CAGCGGCACGAGGCAGAGTC	
CD162	5′ primer: GCGTGGGCTGGGACCTTGTC	168
	3′ primer: TGCCCAGGTGTCCCACAGCT	

### E-selectin and E-selectin ligand quantification

Levels of E-selectin in cell culture supernatants was detected using the Human sE-Selectin/CD62E Quantikine ELISA Kit (Quantikine) according to manufacturer's instructions and using a Thermo Labsystems ELISA plate reader (Multiskan Ascent, Thermo Labsystems).

ECFC cell surface expression of CD44 and CD162 was assessed by flow cytometry using anti-CD162 antibody labeled with Alexa 488 and anti CD44-biotin. The anti-CD162 was labeled in the laboratory using the Alexa Fluor 488 Monoclonal Antibody Labeling Kit (Molecular Probes) whereas anti-CD44 antibody was labeled with biotin using the APEX™ Antibody Labeling Kits (Invitrogen). Streptavidin-PE (Beckman Coulter) was used to detect the biotinylated anti-CD44-antibody. Flow cytometric analysis was performed using a Cyan ADP9 analyzer (Beckman Coulter).

### Statistical analysis

Data are expressed as the mean ± standard deviation. Experiments were performed in triplicate using ECFC cells derived from two separate cord blood samples. Student's *t*-test was used to compare mean values for paired samples. *P<0*.05 was considered statistically significant.

## Results

### ECFC expansion and characterization

**Figure 1:**
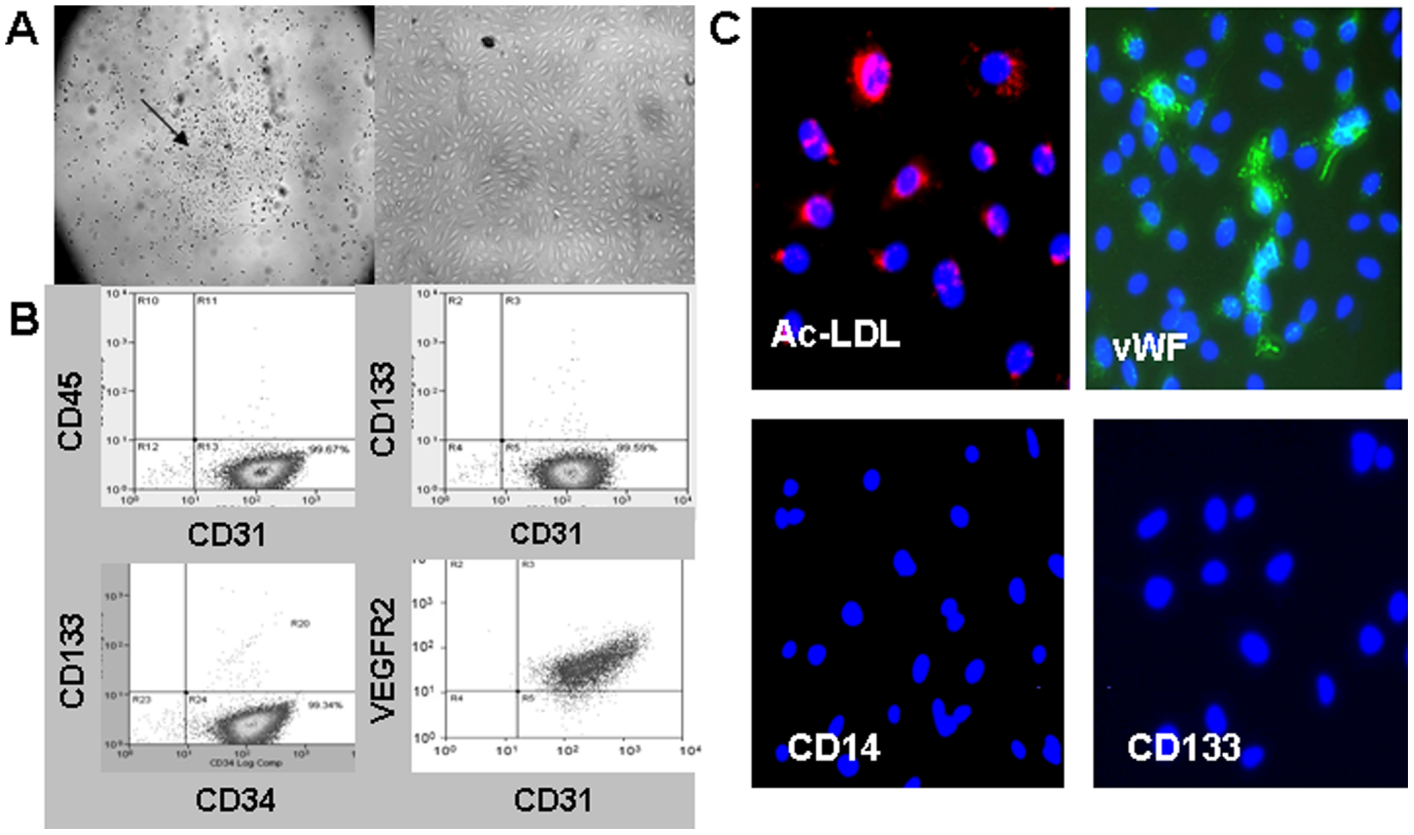
Characterization of Endothelial Colony-Forming Cells (ECFCs). **A)** Typical cell morphology after at least 9 days of culture, 10x magnification (left) and cell morphology after passaging reveals typical cobble stone appearance of a colony with rapid proliferation (see arrow), and 40x magnification (right). **B)** ECFC cells from a typical colony were analyzed by flow cytometry and express CD34 and CD31 surface markers and are negative for CD133 and CD45. **C)** Immunohistochemistry reveals that ECFC cells can take up Ac-LDL and express vWF but not CD14 and CD133.

Mononuclear cells from human umbilical cord blood formed typical cobble stone-like ECFC colonies after a mean of 10 days (range, 9–16 days) in culture under angiogenic conditions (Figure 1A). *Ex vivo* expanded ECFCs were assessed by immunostaining and/or flow cytometry and were positive for the cell surface markers CD34, CD31, VEGFR2 and vWF and were negative for CD45, CD14 and CD133 (Figure 1B and 1C). ECFCs were also able to take up acetylated LDL (Figure 1C).

### E-selectin is released from HUVECs following LPS-induced endotoxic injury

**Figure 2:**
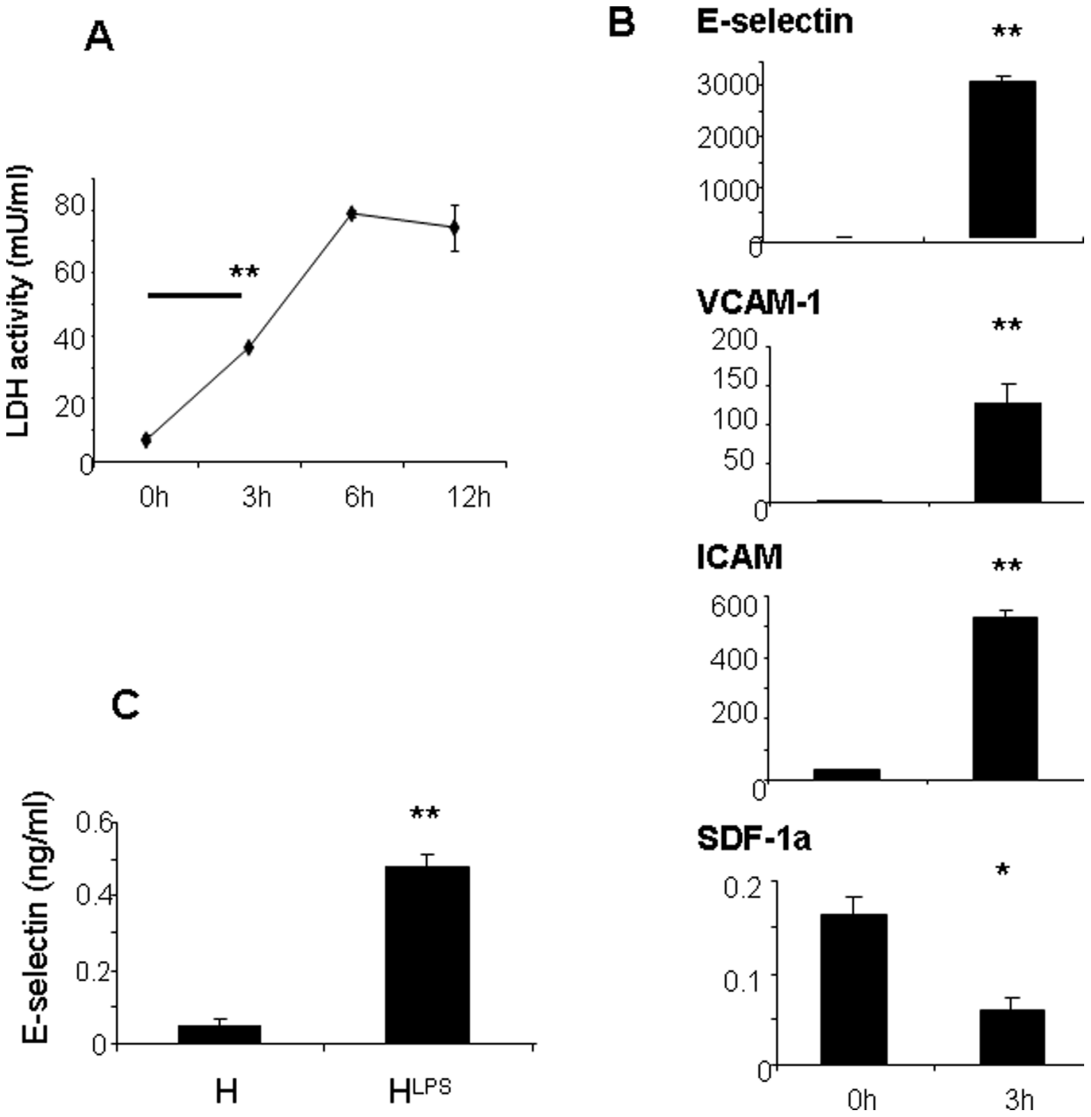
E-selectin, VCAM-1 and ICAM-1 expression is increased in LPS-treated HUVEC cells. **A)** LPS-treated HUVEC cells demonstrate significant cellular injury and increased lactate dehydrogenase (LDH) after 3h (** p<0.01). **B)** Gene expression of E-selectin, VCAM-1, ICAM-1 and SDF-1α induced in HUVECs after endotoxic injury was determined by RT-PCR relative to GAPDH expression (* denotes p<0.05 and ** p<0.01 in comparison with baseline). **C)** E-selectin levels after 12 hours in conditioned media from LPS-treated HUVEC cells (H^LPS^) compared with ECFCs exposed to conditioned media from control HUVEC cells (H) or LPS-treated HUVECs cultured in the presence of anti-CD62E which inhibits soluble E-selectin (H+anti-CD62E)^LPS^, as measured using ELISA.(** p<0.01)

Cellular injury to LPS-treated HUVECs was confirmed by the release of LDH. HUVEC cells demonstrated pronounced injury within 3 hours following exposure to LPS (p<0.01 compared with baseline and uninjured controls) and cellular injury peaked after 6 hours (see Figure 2A). A range of LPS concentrations was tested to obtain the optimal dose of 100 ng/ml to induce cellular injury (see Figure S1). Expression of key genes associated with endothelial injury was performed following LPS treatment of HUVEC cells and included assessment of E-selectin, VCAM-1, ICAM-1 and SDF-1α. Expression level of each gene was determined relative to GAPDH expression (Figure 2B) and also evaluated by the ratio of mean copy number from each time point compared to copy number at baseline (0 hours). The greatest increase in gene expression was observed for E-selectin at all time points assessed (208 ± 9.3-fold increase in expression at 3 hours compared to baseline, p<0.01) while VCAM-1 and ICAM-1 increased 65.1 ± 13.1-fold (p<0.01) and 15.9 ± 0.73-fold (p<0.01) after 3 hours compared to baseline expression levels and SDF-1α expression decreased with a 0.4 ± 0.3-fold change in expression after 3 hours (p<0.05). Notably, SDF-1α mRNA levels in LPS-treated HUVEC cells remained significantly reduced throughout the 12h period of assessment following LPS treatment compared with baseline (p<0.05). Moreover, expression of E-selectin induced by LPS was accompanied by a marked increase in secretion of E-selectin from HUVEC cells with levels in the conditioned media of 0.53 ± 0.07 ng/ml detected by ELISA after 12 hours compared to 0.04 ± 0.007 ng/ml in uninjured control HUVEC cells (p<0.01).

### E-selectin enhances ECFC homing function

**Figure 3:**
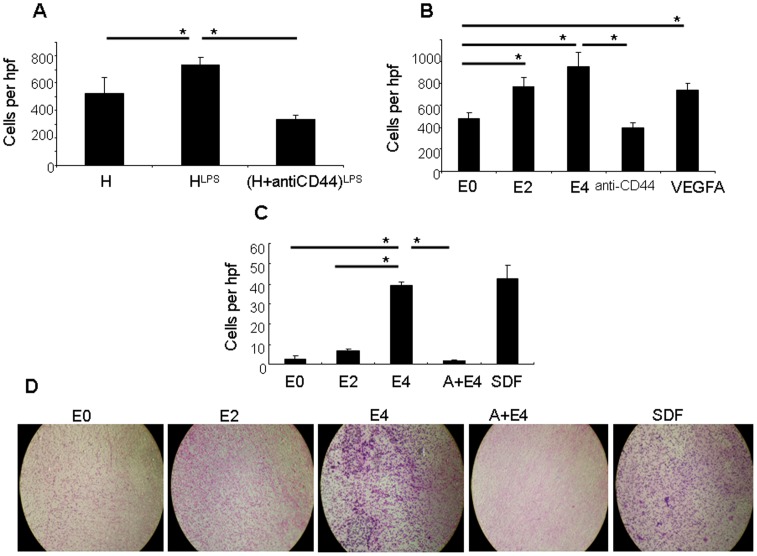
Increased ECFC adhesion and migration following incubation with E-selectin. **A)** ECFC adhesion to fibronectin following exposure to conditioned media from control HUVEC cells (H), from injured HUVEC cells (H^LPS^), and from LPS-treated HUVECs with the addition of anti-CD62E (H+anti-CD62E)^LPS^ and shown as cells per high powered field (hpf) (* p<0.05). **B)** Adhesion of ECFCs to fibronectin following 2 hour incubation with E-selectin at 2 µg/ml (E2) or 4 µg/ml (E4) compared to controls (0 µg/ml, E0), in the presence of anti-CD44 and E-selectin (4 µg/ml) and VEGF-A 100 ng/ml (VEGFA). (* p<0.05). **C)** Migration of ECFCs towards E-selectin in the lower chamber at 2 µg/ml (E2) or 4 µg/ml (E4) compared with controls containing no E-selectin in the lower chamber (E0) or when ECFCs were incubated in the presence of anti-CD44 with E-selectin 4 µg/ml in the lower chamber (A+E4), and to SDF-1a (100 ng/ml) in the lower chamber (SDF). (* p<0.05). **D)** Photgraphs of the underside of porous membranes used in the migration assays described in part C.

ECFC homing function was evaluated using *in vitro* tests of adhesion to the extracellular matrix protein fibronectin (FN) and a Boyden-chamber assay of ECFC migration towards gradients of cytokines associated with endothelial injury. ECFCs were exposed to the conditioned media of HUVEC cells undergoing endotoxic injury or to conditioned media from uninjured HUVEC cells. ECFCs exposed to the conditioned media from injured HUVEC cells demonstrated greater adhesion to fibronectin-coated plastic dishes compared to ECFCs incubated with conditioned media from uninjured cells (730 ± 79 vs. 505 ± 51 attached cells per high powered field (hpf), respectively, p = 0.01). The role of E-selectin in conditioned media from injured HUVEC cells was assessed using anti-CD62E antibodies. Adhesion of ECFCs to fibronectin following incubation with conditioned media from injured HUVECs in the presence of anti-CD62E was markedly reduced to levels below control conditions where ECFCs were incubated with conditioned media from uninjured HUVEC cells (Figure 3A).

The role of E-selectin was examined further by adding exogenous E-selectin to the adhesion buffer used in the assay instead of incubating ECFCs with conditioned media from HUVEC cultures. Exogenous E-selectin increased ECFC adhesion to fibronectin in a concentration-dependent manner. Incubation of 5 × 10^4^ ECFCs with 2 µg/ml and 4 µg/ml E-selectin yielded significantly greater adhesion to fibronectin compared with controls lacking exogenous E-selectin (770 ± 79 and 952 ± 130 adherent cells, respectively, compared with 476 ± 56 adherent cells for controls with 0 µg/ml E-selectin; p<0.01 for all comparisons with control). The impact of E-selectin on adhesion of ECFCs to fibronectin appears similar to 100 ng/ml VEGFA (736 ± 68 adherent cells per field) which has been previously reported to be associated with increased ECFC adhesion [45]. Inhibition of the E-selectin ligand on ECFCs with anti-CD44 antibody abrogated the increase in adhesion to fibronectin observed with 4 µg/ml exogenous E-selectin (390 ± 50 adherent cells per field; p<0.01 compared with ECFCs treated with 4 µg/ml E-selectin and no anti-CD62E antibody) (Figure 3B).

E-selectin was also able to induce greater migration of ECFCs in a concentration-dependent manner. ECFCs plated in the upper part of a Boyden chamber and exposed to 4 µg/ml E-selectin in the lower part had greater migration ratios across the membrane compared to controls with 0 µg/ml E-selectin (39 ± 2.0 vs. 2.5 ± 2.0 migrated cells per hpf; p<0.01). The migration of ECFCs in response to 4 µg/ml E-selectin was similar to ECFC migration towards 100 ng/ml SDF-1α (43 ± 7.0), which has been previously associated with ECFC migration. When ECFCs were treated with anti-CD62E antibody, the migration of ECFCs towards 4 µg/ml E-selectin was reduced to levels observed for controls without E-selectin in the lower chamber (1.5 ± 1.0; p<0.01 in comparison with 4 µg/ml E-selectin) (Figure 3C).

Taken together, these results confirm that E-selectin is upregulated and secreted into the conditioned media by mature endothelial cells undergoing LPS-induced endotoxic injury and E-selectin can facilitate migration of ECFCs and increased adhesion to the extracellular matrix protein fibronectin.

### SDF-1α augments homing function of ECFCs via CXCR4

**Figure 4:**
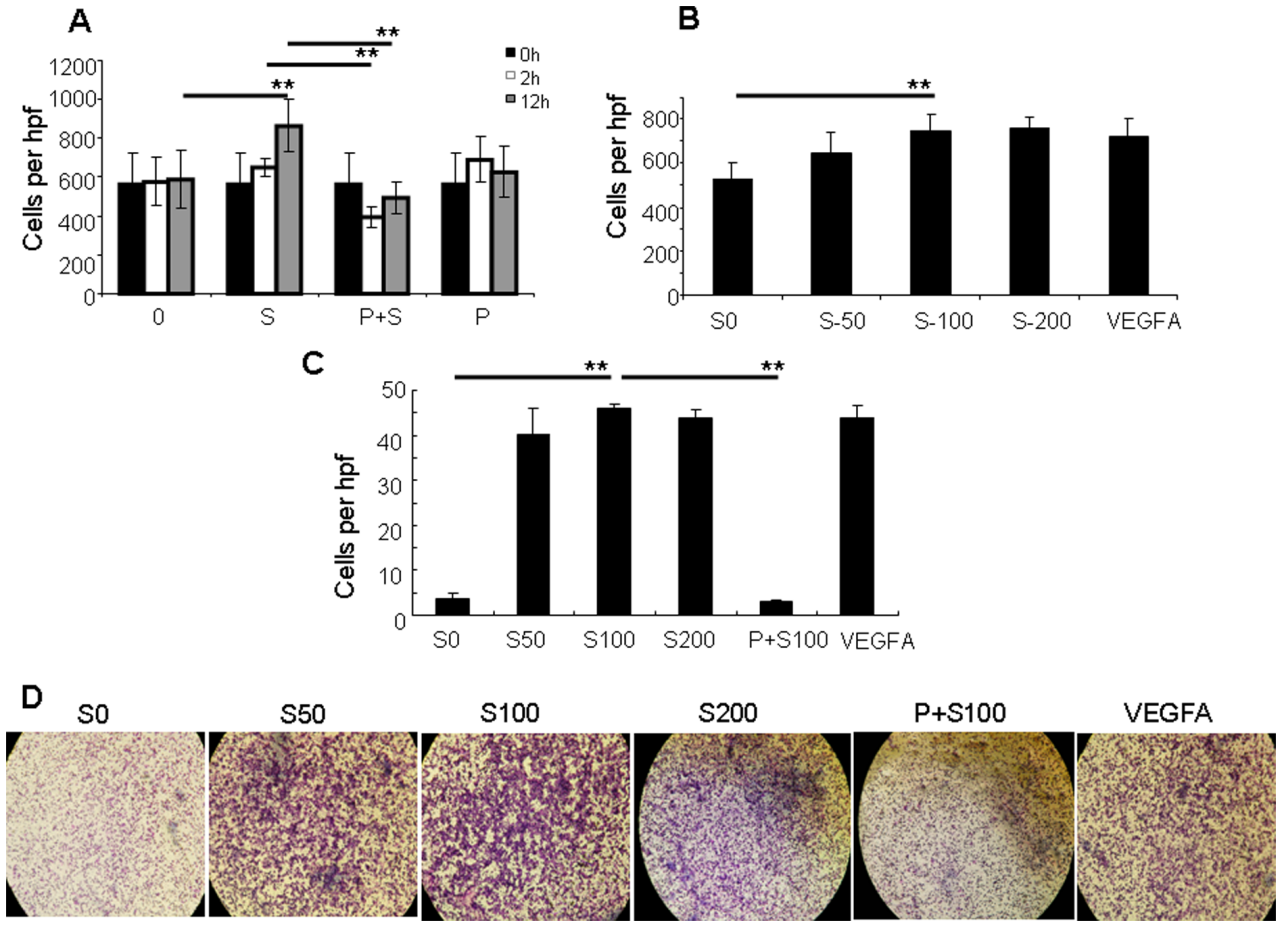
SDF-1α increases adhesion and migration of ECFCs. **A)** Increased adhesion of ECFC cells to fibronectin was observed following incubation with SDF-1α for 12 hours (S) compared with controls (0) (** p<0.01). The increase in adhesion due to SDF-1a was completely abrogated at 2 and 12 hours in the presence of plerixafor, a CXCR-4 antagonist (P+S) (** p<0.01). **B)** Incubating ECFCs with increasing concentrations of SDF-1α (S0  =  0 ng/ml; S50  =  50 ng/ml; S100  =  100 ng/ml; S200  =  200 ng/ml and VEGFA  =  100 ng/ml VEGFA) was associated with increased adhesion to fibronectin. (** p<0.01). **C)** The migration of ECFCs across a porous membrane was enhanced by the presence of SDF-1 (50 – 200 ng/ml) in the lower part of a Boyden chamber, which was inhibited by plerixafor (P+S100). (** p<0.01) **D)** The lower side of the porous membrane can be stained to count the number of migrated cells and calculate per membrane pore. (viewed at 40x).

ECFCs were incubated with exogenous SDF-1α to quantify the impact on adhesion to fibronectin and migration function. ECFCs treated with 100 ng/ml SDF-1α for 12 hours had increased adhesion compared to controls lacking exogenous SDF-1α (865 ± 133 vs. 586 ± 150 adherent cells per hpf; p<0.01). Incubation of ECFCs with the CXCR4 antagonist, plerixafor, attenuated the effect of exogenous SDF-1α (100 ng/ml) on ECFC adhesion after just 2 hours (394 ± 56 with plerixafor vs. 648 ± 47 adherent cells per hpf without plerixafor; p<0.01) and after 12 hours (499 ± 83 vs. 865 ± 133 adherent cells per hpf, respectively; p<0.01). ECFCs treated with plerixafor alone without exogenous SDF-1α had similar adhesion to fibronectin in comparison with control (Figure 4A). The impact of exogenous SDF-1α on the adhesion of ECFCs to fibronectin was concentration dependent up to 100 ng/ml (538 ± 88 adherent cells for 0 ng/ml vs. 647 ± 96 for 50 ng/ml SDF-1α vs. 751 ± 69 for 100 ng/ml; p<0.01) but no further increase in ECFC adhesion was observed at 200 ng/ml SDF-1α (754 ± 44 adherent cells; p>0.05 in comparison to 100 ng/ml). Adhesion of ECFCs treated with 100 ng/ml SDF-1α was similar to 100 ng/ml VEGFA (695 ± 92 adherent cells, p>0.05), which is known to increase ECFC adhesion to fibronectin (Figure 4B).

Migration of ECFCs was increased in response to 100 ng/ml SDF-1α in the lower part of the Boyden chamber compared with controls lacking SDF-1α (46 ± 1.0 vs. 3.5 ± 1.0 cells per hpf; p<0.01) and migration was fully inhibited by plerixafor (3.0 ± 0.0; p<0.01 compared with SDF-1α) (Figure 4C and 4D).

Together, these observations demonstrate that SDF-1α can up-regulate ECFC adhesion and migration via its cell surface receptor CXCR4. Although SDF-1α gene expression is not upregulated in mature endothelial cells undergoing endotoxic injury (Figure 2A), SDF-1α may be produced by other perivascular cell types and/or during other types of endothelial injury and we considered the possible cooperative effects of SDF-1α and E-selectin in the process of vascular repair.

### SDF-1α and E-selectin work synergistically to increase ECFC adhesion

**Figure 5:**
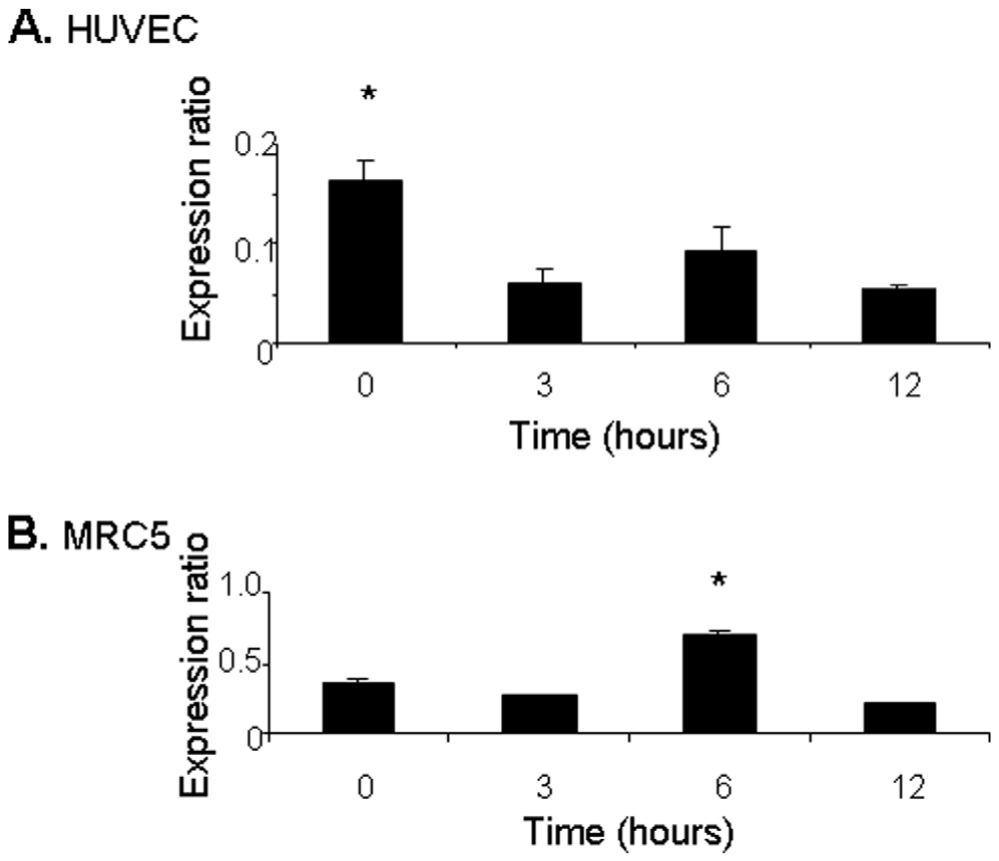
SDF-1α expressed by fibroblasts but not HUVEC cells following LPS treatment. **A)**SDF-1α mRNA expression was assessed after endotoxic injury in HUVECs with an early decrease in expression that was evident at 3 hours and persisted up to 12 hours. (* p>0.05). **B)** Following LPS treatment of MRC5 cells, mRNA expression of SDF-1a is increased after 6h (* p<0.05). Gene expression is reported relative to GAPDH.

**Figure 6:**
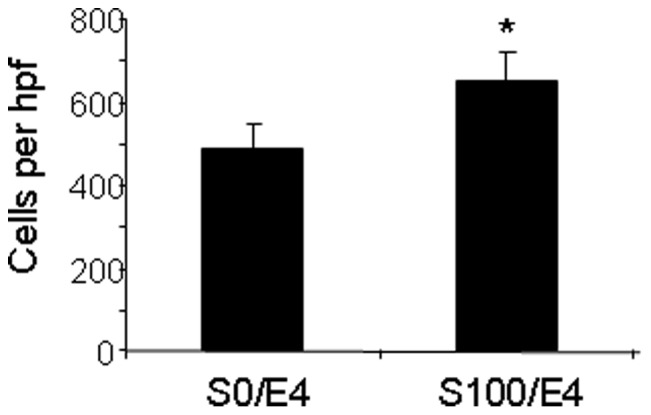
Exogenous SDF-1α augments adhesion of ECFCs to fibronectin. Adhesion of ECFCs to fibronectin after incubation with exogenous recombinant human SDF-1α and recombinant human E-selectin (4 µg/ml) (S100/E4) compared with control ECFCs incubated with E-selectin alone (S0/E4). (* p<0.05).

Our earlier results confirmed that SDF-1α mRNA levels were not increased following endotoxic injury to HUVEC cells (Figure 2A). The possibility of increased SDF-1α expression following endotoxic injury to neighbouring stromal cells was considered. Human lung fibroblasts from the MRC5 cell line were expanded and exposed to endotoxic injury as described above to mimic damage to perivascular cells. SDF-1α expression levels were increased 1.96-fold 6 hours following endotoxic injury to MRC5 cells (P<0.05 compared to baseline expression levels) (Figure 5). Notably, adherence of ECFCs to fibronectin in the presence of E-selectin was further enhanced by pretreatment with SDF-1α (Figure 6).

**Figure 7:**
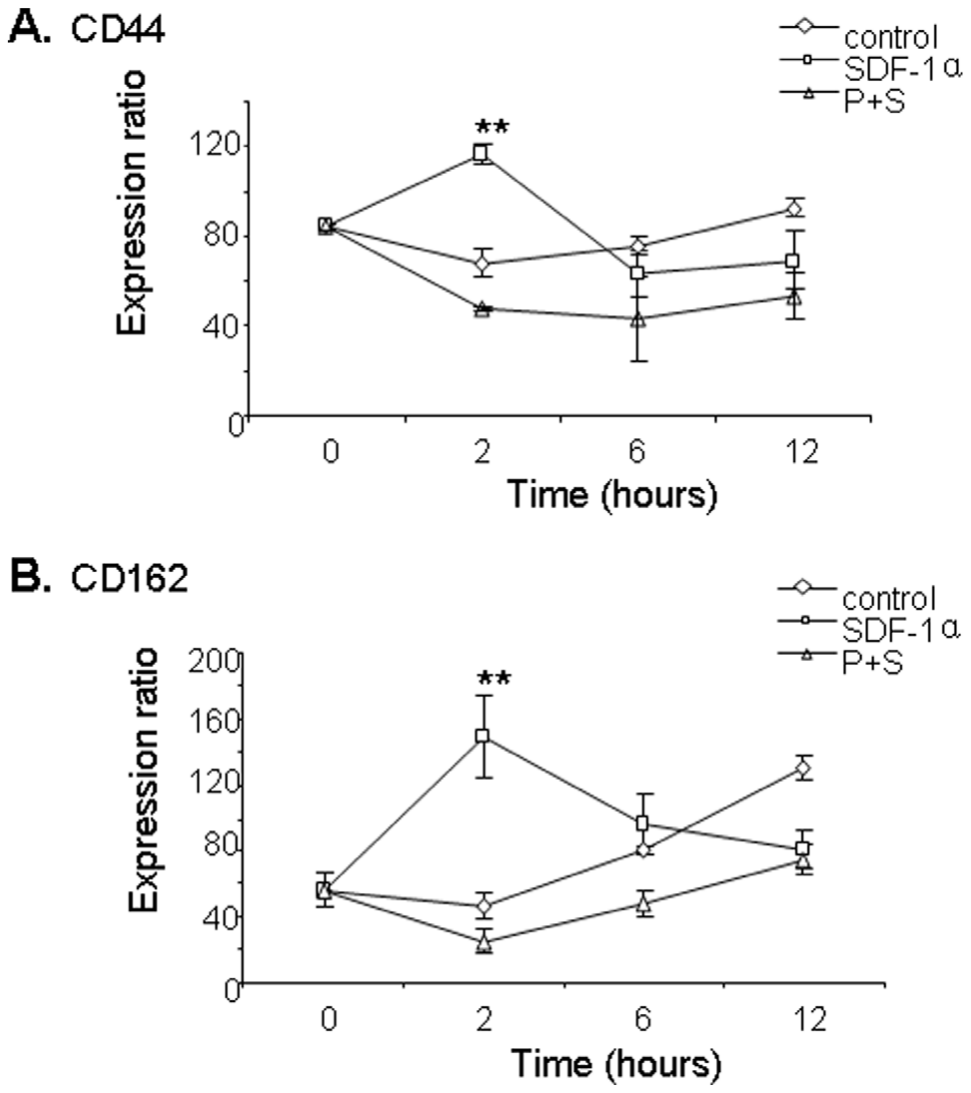
Gene expression of E-selectin ligands on ECFCs treated with SDF-1α. **A)** Expression of CD44 in ECFC cells determined by RT-PCR after treatment with 100 ng/ml SDF-1α (squares), controls without SDF-1α (diamonds) and ECFCs treated wth SDF-1α and plerixafor (triangles). ** p<0.01 comparing expression at 2 hours to 0 hours. **B)** Expression of CD162 on ECFCs. ** p<0.01 comparing expression at 2 hours to 0 hours. Gene expression reported as a ratio compared with GAPDH.

To address potential mechanisms of synergism between SDF-1α and E-selectin, we assessed gene expression of the E-selectin ligands CD44 and CD162 in ECFCs treated with SDF-1α. Both CD44 and CD162 mRNA levels, determined by RT-PCR, increased in ECFCs following treatment with SDF-1α (100 ng/ml) with peak expression at 2 hours following treatment compared to untreated control ECFCs (P<0.05) (Figure 7). Increased CD44 and CD162 gene expression was blocked by plerixafor. Flow cytometric analysis confirmed high levels of CD44 (>80% cells positive) and CD162 (>99% cells positive) on the cell surface of ECFCs but no appreciable change in fluorescence intensity or the percentage of cells that were CD44(+) or CD162(+) following incubation with SDF-1α at 100 ng/ml for 12 hours.

These findings suggest SDF-1α may be released from perivascular cells such as injured fibroblasts and up-regulate expression of E-selectin ligands in ECFCs. Although SDF-1a increases gene expression of CD44 and CD162 and is dependent on CXCR-4, the extent to which increased CD44 and CD162 expression impacts E-selectin mediated adhesion and migration of ECFCs remains unclear.

### Profile of SDF-1α and E-selectin expression in hypoxia and radiation-induced injury

**Figure 8:**
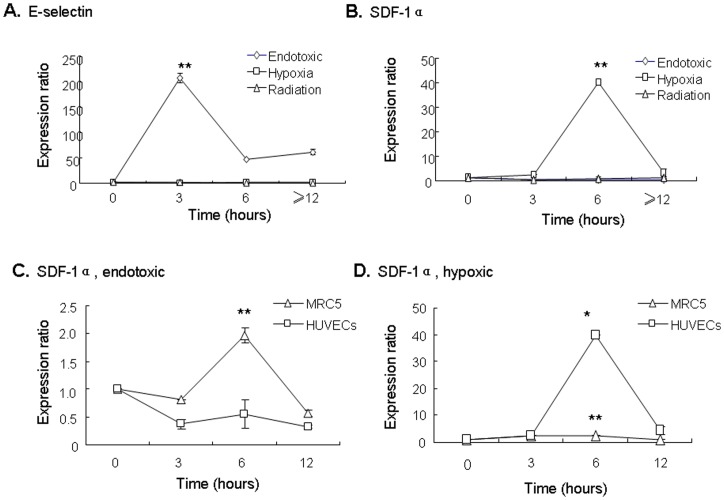
Expression of injury signals is injury-specific and cell-specific. **A)** E-selectin expression in HUVEC cells following endotoxic (diamonds), hypoxic (squares) and radiation injury (triangles). ** p<0.01 for endotoxic injury at 3h compared to 0h. **B)** SDF-1α expression in HUVEC cells following endotoxic (diamonds), hypoxic (squares) or radiation injury (triangles). ** p<0.01 for hypoxic injury at 6h compared to 0h. **C)** SDF-1α expression following endotoxic injury to MRC5 cells (triangles) and HUVEC cells (squares), ** p<0.01 when comparing expression levels at 6h. **D)** SDF-1α expression following hypoxic injury to MRC5 cells (triangles) and HUVEC cells (squares), ** p<0.01 comparing expression levels at 6h; * p<0.05 comparing expression levels at 6 hours in MRC5 cells to 0 hours.

To better understand the potential synergistic effects between SDF-1α and E-selectin in other types of vascular injury, we investigated expression of SDF-1α and E-selectin following radiation-induced and hypoxic injury to HUVECs and MRC5 cells. In contrast to the marked increased in expression after endotoxic injury (208-fold increase compared with baseline, p<0.01), E-selectin expression in HUVEC cells was unchanged after irradiation and hypoxic injury (Figure 8A). As we observed previously following endotoxic injury, SDF-1α expression in HUVEC cells did not change following irradiation but increased by 40-fold by 6 hours following hypoxic injury (p<0.01 compared to baseline) (Figure 8B). In addition to injury-specific patterns of expression, we examined cell-specific expression of E-selectin and SDF-1α. While E-selectin expression was not increased in MRC5 cells after all types of injury examined (data not shown), SDF-1α expression did not increase in HUVEC cells following radiation or endotoxic injury but was markedly increased in MRC5 cells (2.56-fold increase, p<0.05) following endotoxic injury (Figure 8C) and in HUVEC cells (40-fold, p<0.05) following hypoxic injury (Figure 8D).

Our findings highlight important differences in cellular responses to injury depending on the type of injury. In particular, the different profile of SDF-1α and E-selectin expression by endothelial cells and fibroblasts may have significant implications for the synergistic effects on ECFC adhesion and homing function depending on the type of injury.

## Discussion

The results of our study provide new insight on the coordination of vascular repair mediated by ECFCs. E-selectin produced by endothelial cells undergoing endotoxic injury facilitates ECFC adhesion to the extracellular matrix protein fibronectin, and to damaged endothelial cells via E-selectin ligands present on ECFCs. In addition, ECFCs can migrate towards increased E-selectin gradients and may be particularly suited for homing to areas of endotoxic vascular injury. Moreover, our observations suggest a potential mechanism to explain the cooperative effects of SDF-1α and E-selectin in the adhesion and migration function of ECFCs, mediated through CXCR4-dependent expression of the E-selectin ligands CD44 and CD162 on ECFCs. We observed distinct patterns of E-selectin and SDF-1α expression in mature endothelial cells and fibroblasts following different modes of injury, providing new insight regarding potential mechanisms of vascular repair mediated by ECFCs following endothelial damage. Strategies to augment vascular repair using ECFCs should consider the type of endothelial injury and the cooperative effects of SDF-1α and E-selectin on ECFC function.

Repair of endothelial damage is a complex process involving multiple cell types with angiogenic and vascular repair capacity. Hematopoietic cells such as angiogenic monocytes can home to areas of vascular injury [3], [4] and circulating endothelial progenitors have been partially characterized as rare bone marrow-derived or vessel wall-derived precursors of endothelial lineage that can also contribute to vascular repair.[2] ECFCs, however, can be readily expanded from peripheral blood or umbilical cord blood and retain a high proliferative capacity with the ability to incorporate into damaged blood vessels and facilitate vascular repair in several pre-clinical models.[5]-[7] Maximizing their therapeutic potential will require greater insight regarding how they respond to injury signals produced by damaged endothelium. To date, most pre-clinical experimental models have studied ECFC function following hypoxic injury. The role of hypoxia-inducible factors and SDF-1α have been well described in several studies, underscoring the importance of CXCR4 signaling in various populations of endothelial progenitor populations. The role of other injury signals such as E-selectin, however, produced during endotoxic injury and their influence on ECFC function has been less well characterized and potential relationships between E-selectin and SDF-1α on ECFC function have not been previously reported, to the best of our knowledge.

Sepsis remains a major medical challenge, characterized by excessive endotoxic injury to the microvasculature and larger vessels with the risk of extensive perivascular tissue damage and organ dysfunction. Major morbidity and mortality remain a reality for many patients. Several groups have characterized signals produced by activated endothelial cells during sepsis or LPS-induced experimental endotoxic injury, including E-selectin, ICAM-1, other adhesion molecules and cytokines released from injured HUVEC cells. [39], [46]-[48] Our finding that expression of E-selectin, ICAM-1 and VCAM-1 were markedly increased 3h following endotoxic treatment of HUVEC cells is consistent with previous reports.[49] The production of SDF-1α following endotoxic injury of mature endothelial cells, however, has not been specifically reported and in our study, we found SDF-1α expression was not increased by LPS-treatment of HUVEC cells. Perivascular stromal cells such as fibroblasts are present in vascular beds affected by sepsis and may produce injury signals that contribute to the mobilization and coordination of the cellular response involved in vascular repair. In particular, previous reports have identified the production of inflammatory cytokines such as G-CSF, GM-CSF, IL-1, CCL2, and CCL20 produced by lung fibroblasts that contribute to the cytokine storm and capillary leak syndrome that characterizes sepsis.[50] Increased SDF-1α expression, however, has not been described in these previous studies although SDF-1α can be released from activated platelets [51] or from other stromal cells during acute inflammation and sepsis.[52] Our finding that SDF-1α is upregulated in MRC5 cells undergoing endotoxic injury allows us to consider a role for SDF-1α signaling in conjunction with mediators of ECFC function during sepsis-related endothelial injury. The cooperative effects of SDF-1α and E-selectin to increase ECFC adhesion function and to increase ECFC migration towards E-selectin may be partly explained by SDF-1α –mediated expression of E-selectin ligands CD44 and CD162 in ECFCs. The extent to which increased CD44 and CD162 expression impacts E-selectin mediated adhesion and migration of ECFCs requires additional study and a greater understanding of the gene regulatory network influenced by SDF-1α in ECFCs is needed to gain more insight on intracellular signaling pathways involved in ECFC function.

With regards to other modes of endothelial injury, it was interesting to note that both SDF-1α and E-selectin were not increased in HUVEC cells or MRC5 cells following irradiation in our study. One previous report, however, described increased SDF-1α expression after 24 hours in HUVEC cells following 500 cGy radiation [53], suggesting that the kinetics of injury and injury repair mechanisms may be delayed. ECFC-mediated repair of radiation-induced endothelial damage requires further study to better appreciate precise mechanisms of ECFC adhesion and migration in this model.

Other studies addressing the adhesion and migration of endothelial progenitors have been reported but involve several different endothelial progenitor cell types. Shao et *al* [54] reported that 100 ng/ml SDF-1α up-regulates ECFC migration and capillary formation *in vitro*, while Zheng *et al* [55] reported that SDF-1α up-regulates the migration of early outgrowth endothelial progenitors (EPCs) in a concentration-dependent manner and that this migration is inhibited by plerixafor, the CXCR4 antagonist. Interestingly, Liu *et al* [37] reported that SDF-1α can up-regulate the adhesion of early outgrowth EPCs to mature endothelial cells and can increase the expression of E-selectin in mature endothelial cells (HMECs) up to 2.3-fold after 4h. They also observed that SDF-1α -mediated migration of early outgrowth EPCs was E-selectin dependent by up-regulating E-selectin ligands on early EPCs. The use of heterogenous early outgrowth EPCs, which are mostly hematopoietic in origin, makes it difficult to know which cell type is most influenced by SDF-1α in these studies. Further, the therapeutic role of early outgrowth EPCs remains uncertain. The induction of cell surface CD44 on ECFCs by SDF-1α may also explain the increased adhesion to fibronectin observed in our studies as CD44 mediates binding to the COOH-terminal heparin binding domain of fibronectin [56].

With regards to E-selectin production following different types of cellular injury and its effect on endothelial progenitor function, Sermsathanasawadi *et al* [44] reported that enhanced E-selectin expression in irradiated mature endothelial cells augments the adhesion of early outgrowth EPC cells. Oh *et al* [36] have reported on the role of E-selectin in the adhesion, migration and *in vitro* capillary forming ability of early outgrowth EPCs, which can be blocked by anti-E-selectin antibody. Moreover, E-selectin augments early outgrowth EPC-induced vasculogenesis in ischemic limbs using in vivo animal models.[37] Combined with our observations concerning ECFC function, the upregulation of E-selectin expression appears to be a central mechanism used by various types of endothelial progenitor cells in a range of injury models involving different types of injury and different tissue types.

Our research provides new potential insight on the importance of crosstalk between cellular injury signals produced within the injured microvascular microenvironment. In particular, our results provide an important foundation regarding the interaction of SDF-1α and E-selectin in vascular repair using an in vitro model of endotoxin injury in a homogeneous population of ECFC endothelial progenitors. Studies in vascular repair will need to consider cell specific and injury-specific signals and their effects on homogenous cell populations of particular clinical relevance to translate cell-mediated vascular repair strategies into clinical reality. In addition, our studies will require validation in animal models of acute tissue injury to validate our observations and to further develop potential strategies designed to facilitate vascular repair.

## Supporting Information

Figure S1 **Cell injury in HUVEC cells induced by LPS.** LDH activity is reported in conditioned media of cultured HUVEC cells incubated with LPS at varying concentrations. LDH measurements were performed in accordance with manufacturer's instructions and provide an estimate of cellular damage (described in Methods section).
(TIF)

## References

[1] Asahara T, Murohara T, Sullivan A, Silver M, van der Zee R, et al (1997) Isolation of putative progenitor endothelial cells for angiogenesis. *Science* 275: 964–967. 9020076 10.1126/science.275.5302.964

[2] Strunk D (2011) Endothelial progenitor cells: quod erat demonstrandum? *Curr Pharm Des* 17: 3245–3251. 21919881 10.2174/138161211797904127

[3] Rehman J, Li J, Orschell CM, March KL (2003) Peripheral blood “endothelial progenitor cells” are derived from monocyte/macrophages and secrete angiogenic growth factors. *Circulation* 107: 1164–1169. 12615796 10.1161/01.cir.0000058702.69484.a0

[4] Rohde E, Bartmann C, Schallmoser K, Reinisch A, Lanzer G, et al (2007) Immune cells mimic the morphology of endothelial progenitor colonies in vitro. *Stem Cells* 25: 1746–1752. 17395771 10.1634/stemcells.2006-0833

[5] Lin Y, Weisdorf DJ, Solovey A, Hebbel RP (2000) Origins of circulating endothelial cells and endothelial outgrowth from blood. *J Clin Invest* 105: 71–77. 10619863 10.1172/JCI8071 PMC382587

[6] Hur J, Yoon CH, Kim HS, Choi JH, Kang HJ, et al (2004) Characterization of two types of endothelial progenitor cells and their different contributions to neovasculogenesis. *Arterioscler Thromb Vasc Biol* 24: 288–293. 14699017 10.1161/01.ATV.0000114236.77009.06

[7] Yamaguchi J, Kusano KF, Masuo O, Kawamoto A, Silver M, et al (2003) Stromal cell-derived factor-1 effects on ex vivo expanded endothelial progenitor cell recruitment for ischemic neovascularization. *Circulation* 107: 1322–1328. 12628955 10.1161/01.cir.0000055313.77510.22

[8] Massa M, Rosti V, Ferrario M, Campanelli R, Ramajoli I, et al (2005) Increased circulating hematopoietic and endothelial progenitor cells in the early phase of acute myocardial infarction. *Blood* 105: 199–206. 15345590 10.1182/blood-2004-05-1831

[9] Sobrino T, Hurtado O, Moro MA, Rodriguez-Yanez M, Castellanos M, et al (2007) The increase of circulating endothelial progenitor cells after acute ischemic stroke is associated with good outcome. *Stroke* 38: 2759–2764. 17761925 10.1161/STROKEAHA.107.484386

[10] Lemoli RM, Catani L, Talarico S, Loggi E, Gramenzi A, et al (2006) Mobilization of bone marrow-derived hematopoietic and endothelial stem cells after orthotopic liver transplantation and liver resection. *Stem Cells* 24: 2817–2825. 16931769 10.1634/stemcells.2006-0333

[11] Inoue T, Sata M, Hikichi Y, Sohma R, Fukuda D, et al (2007) Mobilization of CD34-positive bone marrow-derived cells after coronary stent implantation: impact on restenosis. *Circulation* 115: 553–561. 17261663 10.1161/CIRCULATIONAHA.106.621714

[12] Nonaka-Sarukawa M, Yamamoto K, Aoki H, Nishimura Y, Tomizawa H, et al (2007) Circulating endothelial progenitor cells in congestive heart failure. *Int J Cardiol* 119: 344–348. 17070610 10.1016/j.ijcard.2006.07.191

[13] Egan CG, Lavery R, Caporali F, Fondelli C, Laghi-Pasini F, et al (2008) Generalised reduction of putative endothelial progenitors and CXCR4-positive peripheral blood cells in type 2 diabetes. *Diabetologia* 51: 1296–1305. 18286257 10.1007/s00125-008-0939-6

[14] Asahara T, Takahashi T, Masuda H, Kalka C, Chen D, et al (1999) VEGF contributes to postnatal neovascularization by mobilizing bone marrow-derived endothelial progenitor cells. *EMBO J* 18: 3964–3972. 10406801 10.1093/emboj/18.14.3964 PMC1171472

[15] Orlic D, Kajstura J, Chimenti S, Limana F, Jakoniuk I, et al (2001) Mobilized bone marrow cells repair the infarcted heart, improving function and survival. *Proc Natl Acad Sci U S A* 98: 10344–10349. 11504914 10.1073/pnas.181177898 PMC56963

[16] Takahashi T, Kalka C, Masuda H, Chen D, Silver M, et al (1999) Ischemia- and cytokine-induced mobilization of bone marrow-derived endothelial progenitor cells for neovascularization. *Nat Med* 5: 434–438. 10202935 10.1038/7434

[17] Bahlmann FH, DeGroot K, Duckert T, Niemczyk E, Bahlmann E, et al (2003) Endothelial progenitor cell proliferation and differentiation is regulated by erythropoietin. *Kidney Int* 64: 1648–1652. 14531796 10.1046/j.1523-1755.2003.00279.x

[18] Lipsic E, van der Meer P, Voors AA, Westenbrink BD, van den Heuvel AF, et al (2006) A single bolus of a long-acting erythropoietin analogue darbepoetin alfa in patients with acute myocardial infarction: a randomized feasibility and safety study. *Cardiovasc Drugs Ther* 20: 135–141. 16761193 10.1007/s10557-006-7680-5

[19] Shepherd RM, Capoccia BJ, Devine SM, Dipersio J, Trinkaus KM, et al (2006) Angiogenic cells can be rapidly mobilized and efficiently harvested from the blood following treatment with AMD3100. *Blood* 108: 3662–3667. 16912220 10.1182/blood-2006-06-030577 PMC1895468

[20] Hristov M, Zernecke A, Liehn EA, Weber C (2007) Regulation of endothelial progenitor cell homing after arterial injury. *Thromb Haemost* 98: 274–277. 17721606

[21] Wysocki SJ, Zheng MH, Smith A, Lamawansa MD, Iacopetta BJ, et al (1996) Monocyte chemoattractant protein-1 gene expression in injured pig artery coincides with early appearance of infiltrating monocyte/macrophages. *J Cell Biochem* 62: 303–313. 8872602 10.1002/(sici)1097-4644(199609)62:3<303::aid-jcb1>3.0.co;2-v

[22] Duan H, Cheng L, Sun X, Wu Y, Hu L, et al (2006) LFA-1 and VLA-4 involved in human high proliferative potential-endothelial progenitor cells homing to ischemic tissue. *Thromb Haemost* 96: 807–815. 17139377

[23] Chavakis E, Aicher A, Heeschen C, Sasaki K, Kaiser R, et al (2005) Role of beta2-integrins for homing and neovascularization capacity of endothelial progenitor cells. *J Exp Med* 201: 63–72. 15623573 10.1084/jem.20041402 PMC2212777

[24] Hristov M, Zernecke A, Bidzhekov K, Liehn EA, Shagdarsuren E, et al (2007) Importance of CXC chemokine receptor 2 in the homing of human peripheral blood endothelial progenitor cells to sites of arterial injury. *Circ Res* 100: 590–597. 17272812 10.1161/01.RES.0000259043.42571.68

[25] Massberg S, Konrad I, Schurzinger K, Lorenz M, Schneider S, et al (2006) Platelets secrete stromal cell-derived factor 1alpha and recruit bone marrow-derived progenitor cells to arterial thrombi in vivo. *J Exp Med* 203: 1221–1233. 16618794 10.1084/jem.20051772 PMC2121205

[26] Huo Y, Weber C, Forlow SB, Sperandio M, Thatte J, et al (2001) The chemokine KC, but not monocyte chemoattractant protein-1, triggers monocyte arrest on early atherosclerotic endothelium. *J Clin Invest* 108: 1307–1314. 11696575 10.1172/JCI12877 PMC209441

[27] Smith C, Damas JK, Otterdal K, Oie E, Sandberg WJ, et al (2006) Increased levels of neutrophil-activating peptide-2 in acute coronary syndromes: possible role of platelet-mediated vascular inflammation. *J Am Coll Cardiol* 48: 1591–1599. 17045893 10.1016/j.jacc.2006.06.060

[28] Foubert P, Silvestre JS, Souttou B, Barateau V, Martin C, et al (2007) PSGL-1-mediated activation of EphB4 increases the proangiogenic potential of endothelial progenitor cells. *J Clin Invest* 117: 1527–1537. 17510705 10.1172/JCI28338 PMC1866248

[29] Walter DH, Cejna M, Diaz-Sandoval L, Willis S, Kirkwood L, et al (2004) Local gene transfer of phVEGF-2 plasmid by gene-eluting stents: an alternative strategy for inhibition of restenosis. *Circulation* 110: 36–45. 15210598 10.1161/01.CIR.0000133324.38115.0A

[30] Togel F, Isaac J, Hu Z, Weiss K, Westenfelder C (2005) Renal SDF-1 signals mobilization and homing of CXCR4-positive cells to the kidney after ischemic injury. *Kidney Int* 67: 1772–1784. 15840024 10.1111/j.1523-1755.2005.00275.x

[31] Kollet O, Shivtiel S, Chen YQ, Suriawinata J, Thung SN, et al (2003) HGF, SDF-1, and MMP-9 are involved in stress-induced human CD34+ stem cell recruitment to the liver. *J Clin Invest* 112: 160–169. 12865405 10.1172/JCI17902 PMC164291

[32] Ponomaryov T, Peled A, Petit I, Taichman RS, Habler L, et al (2000) Induction of the chemokine stromal-derived factor-1 following DNA damage improves human stem cell function. *J Clin Invest* 106: 1331–1339. 11104786 10.1172/JCI10329 PMC381461

[33] Hattori K, Heissig B, Tashiro K, Honjo T, Tateno M, et al (2001) Plasma elevation of stromal cell-derived factor-1 induces mobilization of mature and immature hematopoietic progenitor and stem cells. *Blood* 97: 3354–3360. 11369624 10.1182/blood.v97.11.3354

[34] Heissig B, Hattori K, Dias S, Friedrich M, Ferris B, et al (2002) Recruitment of stem and progenitor cells from the bone marrow niche requires MMP-9 mediated release of kit-ligand. *Cell* 109: 625–637. 12062105 10.1016/s0092-8674(02)00754-7 PMC2826110

[35] Shen L, Gao Y, Qian J, Wu Y, Zhou M, et al (2012) The role of SDF-1alpha/Rac pathway in the regulation of endothelial progenitor cell polarity; homing and expression of Rac1, Rac2 during endothelial repair. *Mol Cell Biochem* 365: 1–7. 21964561 10.1007/s11010-011-1083-z

[36] Oh IY, Yoon CH, Hur J, Kim JH, Kim TY, et al (2007) Involvement of E-selectin in recruitment of endothelial progenitor cells and angiogenesis in ischemic muscle. *Blood* 110: 3891–3899. 17699745 10.1182/blood-2006-10-048991

[37] Liu ZJ, Tian R, An W, Zhuge Y, Li Y, et al (2010) Identification of E-selectin as a novel target for the regulation of postnatal neovascularization: implications for diabetic wound healing. *Ann Surg* 252: 625–634. 20881769 10.1097/SLA.0b013e3181f5a079 PMC3391745

[38] Critser PJ, Yoder MC (2010) Endothelial colony-forming cell role in neoangiogenesis and tissue repair. *Curr Opin Organ Transplant* 15: 68–72. 19898235 10.1097/MOT.0b013e32833454b5 PMC2880951

[39] Magder S, Neculcea J, Neculcea V, Sladek R (2006) Lipopolysaccharide and TNF-alpha produce very similar changes in gene expression in human endothelial cells. *J Vasc Res* 43: 447–461. 16921252 10.1159/000095162

[40] Shioiri T, Muroi M, Hatao F, Nishida M, Ogawa T, et al (2009) Caspase-3 is activated and rapidly released from human umbilical vein endothelial cells in response to lipopolysaccharide. *Biochim Biophys Acta* 1792: 1011–1018. 19559790 10.1016/j.bbadis.2009.06.006

[41] Maeng YS, Choi HJ, Kwon JY, Park YW, Choi KS, et al (2009) Endothelial progenitor cell homing: prominent role of the IGF2-IGF2R-PLCbeta2 axis. *Blood* 113: 233–243. 18832656 10.1182/blood-2008-06-162891

[42] Youn SW, Lee SW, Lee J, Jeong HK, Suh JW, et al (2011) COMP-Ang1 stimulates HIF-1alpha-mediated SDF-1 overexpression and recovers ischemic injury through BM-derived progenitor cell recruitment. *Blood* 117: 4376–4386. 21200018 10.1182/blood-2010-07-295964

[43] Riederer I, Sievert W, Eissner G, Molls M, Multhoff G (2010) Irradiation-induced up-regulation of HLA-E on macrovascular endothelial cells confers protection against killing by activated natural killer cells. *PLoS One* 5: e15339. 21179573 10.1371/journal.pone.0015339 PMC3002963

[44] Sermsathanasawadi N, Ishii H, Igarashi K, Miura M, Yoshida M, et al (2009) Enhanced adhesion of early endothelial progenitor cells to radiation-induced senescence-like vascular endothelial cells in vitro. *J Radiat Res (Tokyo)* 50: 469–475. 19628926 10.1269/jrr.09036

[45] Liu ZJ, Velazquez OC (2008) Hyperoxia, endothelial progenitor cell mobilization, and diabetic wound healing. *Antioxid Redox Signal* 10: 1869–1882. 18627349 10.1089/ars.2008.2121 PMC2638213

[46] Bevilacqua MP, Pober JS, Mendrick DL, Cotran RS, Gimbrone MA Jr (1987) Identification of an inducible endothelial-leukocyte adhesion molecule. *Proc Natl Acad Sci U S A* 84: 9238–9242. 2827173 10.1073/pnas.84.24.9238 PMC299728

[47] Bevilacqua MP (1993) Endothelial-leukocyte adhesion molecules. *Annu Rev Immunol* 11: 767–804. 8476577 10.1146/annurev.iy.11.040193.004003

[48] Lush CW, Cepinskas G, Kvietys PR (2000) LPS tolerance in human endothelial cells: reduced PMN adhesion, E-selectin expression, and NF-kappaB mobilization. *Am J Physiol Heart Circ Physiol* 278: H853–861. 10710354 10.1152/ajpheart.2000.278.3.H853

[49] Beck GC, Rafat N, Brinkkoetter P, Hanusch C, Schulte J, et al (2006) Heterogeneity in lipopolysaccharide responsiveness of endothelial cells identified by gene expression profiling: role of transcription factors. *Clin Exp Immunol* 143: 523–533. 16487252 10.1111/j.1365-2249.2006.03005.x PMC1809605

[50] Perros F, Lambrecht BN, Hammad H (2011) TLR4 signalling in pulmonary stromal cells is critical for inflammation and immunity in the airways. *Respir Res* 24: 125. 10.1186/1465-9921-12-125 PMC3189122 21943186

[51] Stellos K, Gawaz M (2007) Platelet interaction with progenitor cells: potential implications for regenerative medicine. *Thromb Haemost* 98: 922–929. 18000594 10.1160/th07-02-0147

[52] Mizuno K, Matsuyama W, Mitsuyama H, Watanabe M, Higashimoto I, et al (2005) Clinical investigation: increased serum stromal derived factor 1 alpha levels in pulmonary tuberculosis. *Clin Exp Immunol* 139: 490–497. 15730395 10.1111/j.1365-2249.2005.02721.x PMC1809316

[53] Lerman OZ, Greives MR, Singh SP, Thanik VD, Chang CC, et al (2010) Low-dose radiation augments vasculogenesis signaling through HIF-1-dependent and -independent SDF-1 induction. *Blood* 116: 3669–3676. 20631377 10.1182/blood-2009-03-213629

[54] Shao H, Tan Y, Eton D, Yang Z, Uberti MG (2008) Statin and stromal cell-derived factor-1 additively promote angiogenesis by enhancement of progenitor cells incorporation into new vessels. *Stem Cells* 26: 1376–1384. 18308946 10.1634/stemcells.2007-0785

[55] Zheng H, Fu G, Dai T, Huang H (2007) Migration of endothelial progenitor cells mediated by stromal cell-derived factor-1alpha/CXCR4 via PI3K/Akt/eNOS signal transduction pathway. *J Cardiovasc Pharmacol* 50: 274–280. 17878755 10.1097/FJC.0b013e318093ec8f

[56] Jalkanen S, Jalkanen M (1992) Lymphocyte CD44 binds the COOH-terminal heparin-binding domain of fibronectin. *J Cell Biol* 116: 817–825. 1730778 10.1083/jcb.116.3.817 PMC2289325

