# Supplementary figures and images for: E-Selectin Mediated Adhesion and Migration of Endothelial Colony Forming Cells Is Enhanced by SDF-1α/CXCR4
Source: PLoS One. 2013 Apr 2;8(4):e60890. doi: 10.1371/journal.pone.0060890 (PMC3614942; doi:10.1371/journal.pone.0060890)

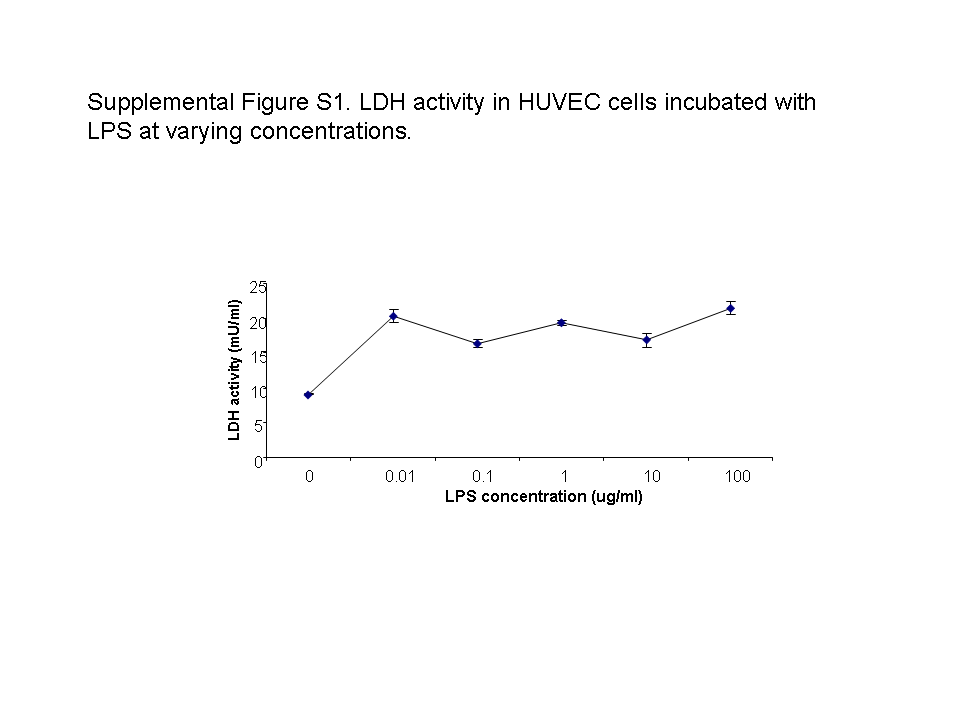

Supplement: Figure S1 — Cell injury in HUVEC cells induced by LPS. LDH activity is reported in conditioned media of cultured HUVEC cells incubated with LPS at varying concentrations. LDH measurements were performed in accordance with manufacturer's instructions and provide an estimate of cellular damage (described in Methods section). (TIF) [file pone.0060890.s001.tif]
